# ‘Personal Health Surveillance’: The Use of mHealth in Healthcare Responsibilisation

**DOI:** 10.1093/phe/phab013

**Published:** 2021-05-16

**Authors:** Ben Davies

**Affiliations:** Uehiro Centre for Practical Ethics, University of Oxford

## Abstract

There is an ongoing increase in the use of mobile health (mHealth) technologies that patients can use to monitor health-related outcomes and behaviours. While the dominant narrative around mHealth focuses on patient empowerment, there is potential for mHealth to fit into a growing push for patients to take personal responsibility for their health. I call the first of these uses ‘medical monitoring’, and the second ‘personal health surveillance’. After outlining two problems which the use of mHealth might seem to enable us to overcome—fairness of burdens and reliance on self-reporting—I note that these problems would only really be solved by unacceptably comprehensive forms of personal health surveillance which applies to all of us at all times. A more plausible model is to use personal health surveillance as a last resort for patients who would otherwise independently qualify for responsibility-based penalties. However, I note that there are still a number of ethical and practical problems that such a policy would need to overcome. The prospects of mHealth enabling a fair, genuinely cost-saving policy of patient responsibility are slim.

## Mobile Health, Surveillance and Two Challenges for Responsibilisation

Technological advances are providing increasing ability to monitor health outcomes and health-related behaviours outside traditional clinical settings and relationships. Patients can self-administer tests for blood sugars (Cvrkel, 2018; Istepanian and Al-anzi, 2018); oxygen saturation (Pantelopoulos and Bourbakis, 2010); blood pressure (Weber *et al.*, 2012); heart rate (Chow *et al.*, 2016: 804); mood (Harrison *et al.*, 2011); and neurological function (Behar *et al.*, 2019). We can monitor health-related behaviours more easily (Sharon, 2017): wearable technologies can help monitor activity levels and diet (Connelly *et al.*, 2013); alcohol consumption (Cohn *et al.*, 2011); and medication use (Cavoukian *et al.*, 2010; Martani and Starke, 2019: 251), as well as providing mental health services (Martinez-Martin and Kreitmair, 2018). Collectively, these technologies are known as mobile health (mHealth) (WHO Global Observatory for eHealth and World Health Organization, 2011). mHealth is generally defined as a subcategory of ‘e-health’ (Chatzipavlou *et al.*, 2016: 1), which encompasses the general use of information and communications technologies for health (WHO | eHealth, n.d.). mHealth includes applications on mobile phones as well as more direct monitoring of patient health indicators such as wearable monitors and at-home testing kits whose results can be transmitted by patients to medical professionals (DiStefano and Schmidt, 2016).

mHealth has the potential to facilitate two functions. First, it may allow us to monitor our bodily *processes* which, while affected by behaviour, are not under direct control (Lupton, 2013, 2019). If a patient knows broadly which behaviours affect the relevant processes, they can attempt to indirectly moderate their health. mHealth may thus relocate routine health monitoring from explicitly medical settings to the home, workplace and wider world, which Swan (2012) describes as an ‘institutional recasting’ of healthcare (see also Carter *et al.*, 2015). This can be seen positively as ‘shifting (health management) into the hands of empowered patients’ (an ideal reported, critically, by Ruckenstein and Schüll, 2017: 262), liberating them from time-costly medical appointments (Topol, 2015), or more negatively as an over-medicalization of previously more carefree spaces.

Second, mHealth may facilitate monitoring of *behaviours* that affect our health, but which are difficult to track unaided, and about which we are wont to self-deceive. I tell my doctor that I stick to the UK government’s guideline of 14 units of alcohol per week. Perhaps I believe this to be true; judging my unit intake requires a relatively complicated calculation. The relative complexity of keeping tabs on an enjoyable activity facilitates my reluctance to confront the truth about my alcohol consumption. An app that calculates the units for me might help me follow the guidelines.

We might therefore see mHealth as a way for patients to take back control over their health from institutional medicine, saving time and effort by reducing unnecessary contact with medical professionals. Not coincidentally, optimists might see mHealth as promising public spending savings at no cost to public health. Finally, the data generated by the use of mHealth has the potential to feed into public health research.

Yet mHealth could be integrated into healthcare in another way, using technological monitoring to increase the role of individual responsibility not only as a method of empowering patients, but also to hold them accountable as users of public resources. For clarity between these two uses of mHealth devices I use the term ‘medical monitoring’ for more standard current uses of monitoring devices, e.g. allowing patients to access data about themselves. When it comes to using mHealth to enforce responsibility, I will use the term ‘personal health surveillance’. The same device or app can therefore be used in either monitoring or surveillance. As far as I am aware, this distinction has not previously been explicitly discussed in work on mHealth.

The use of mHealth by some insurance companies (Shemkus, 2015; Lupton, 2016: 164; Henkel *et al.*, 2018; O’Neill, 2018; Martani *et al.*, 2019) offers insight into the possible institutional uses of mHealth. While most companies currently use mHealth technology by offering positive rewards to those who achieve particular targets, Raber *et al.* (2019: 1767–8) note that these systems ‘could be used by insurers in the future to penalize users’. Similarly, where private employers are responsible for part of employees’ health insurance costs, there have been attempts by some to mandate employees’ health-related behaviours through the use of mHealth (Lupton, 2016; Nissenbaum and Patterson, 2016: 84, 88; Barlin, 2018). Writing just over a decade ago, Hendrix and Buck (2009: 466) describe how ‘employers have begun to implement increasingly aggressive wellness programs that provide incentives to employees who meet certain health standards, while creating disincentives for those who fail to meet the standards’.

My central focus in this article is on the potential for the state to follow suit. The idea of responsibility is a familiar theme in publicly funded health, with many jurisdictions either implementing or considering measures which would increase costs or affect access to care for those who are suitably responsible for their ill health (Schmidt, 2007, 2009; Hancock, n.d.; Ter Meulen and Maarse, 2009), with Lupton (2016: 155) noting how Anglophone countries have retained a focus on personal responsibility that developed in the mid-20th century, with a ‘renewed emphasis on lifestyle change’. The possibility of responsibility-centred rationing is not an abstract possibility, with Pillutla *et al.* (2018: 1) noting recent local proposals in the UK to ‘restrict elective surgery for patients who either smoke or are obese’.

When health costs are borne partly or wholly by the state, it is not a significant leap to think that where insurance companies and private employers lead, public health systems could be tempted to follow. At one extreme is the widespread use of data for citizen tracking currently operating in China (Botsman, 2017). Yet even if such a comprehensive system of surveillance seems unlikely in more democratic states, the use of mHealth may seem to ‘close the loophole of practical enforceability’ when it comes to judgements of personal responsibility (Martani and Starke, 2019: 241).^1^

As mHealth grows in both private and public usage, particularly in the context of increased political and social focus on personal responsibility, it is important to consider the implications of using mHealth to enforce responsibilisation. In doing so, I remain neutral for the sake of this article about whether it is legitimate to hold people responsible for their health by imposing additional burdens on their access to care when their poor health is suitably due to their own free choices. These burdens may range from the severe (denial of care) to the mild (some additional co-payments).

There are a number of arguments for and against the idea of healthcare responsibilisation. Some ‘luck egalitarians’, for instance, consider inequalities that individuals cannot avoid to be unjust, but do not necessarily condemn inequalities which reflect exercises of responsibility (e.g. Arneson, 1999; Dworkin, 2000: 77–8; Vallentyne, 2002; Lippert-Rasmussen, 2016);^2^ while not all luck egalitarians apply this view directly to real-world healthcare, some do (Roemer, 1993; Cappelen and Norheim, 2005; Segall, 2010; Le Grand, 2013: 303; Albertsen and Knight, 2015; Albertsen, 2020), though generally not in anything like the simplistic manner imagined by critics. Others have argued from alternative perspectives that responsibility may be a reasonable part of any healthcare system (Buyx, 2008; Savulescu, 2018).

Others regard the luck egalitarian stance as excessively ‘harsh’ (Fleurbaey, 1995; Anderson, 1999; Voigt, 2007; Venkatapuram, 2011, 198); see the practical aim of holding people responsible as inappropriately focused on a small section of our choices (Minkler, 1999; Wikler, 2002; Sharkey and Gillam, 2010; Friesen, 2018); criticise a reframing of social problems as individual ones (Ayo, 2012; Lupton, 2012); or doubt our ability to appropriately take responsibility for our health (Levy, 2018). Yet it is of independent value to demonstrate, as I hope to do, that *even if* we grant the legitimacy of holding people responsible in some cases for their poor health, it is very difficult to justify the use of mHealth technologies for enforcing this.

I will shortly outline an initial ‘optimistic’ case for how mHealth might indeed ‘close the loop’ of enforceability for personal responsibility (e.g. Swan, 2012; Wiederhold, 2012; Topol, 2015) before going on to raise a number of ethical and practical challenges. Before doing so, however, it is worth commenting briefly on an area of public health policy which I will not discuss in detail, but which has tangential relevance to this issue.^3^ This is the issue of traditional public health surveillance for the purposes of controlling infectious disease. This issue will be familiar to many because of the (at the time of writing) ongoing COVID-19 pandemic. Infectious disease surveillance obviously predates this crisis and is primarily justified by the potential for exponential escalation and significant harm (Gilbert *et al.*, 2019: 176).

Fairchild *et al.* (2008: 30) outline a traditional understanding of infectious disease surveillance, as ‘the ongoing, name-based reporting of cases of disease to state and local health departments’. However, others (e.g. Samerski, 2018: 1; Mello and Wang, 2020: 951) note the growing influence of mHealth in potentially more proactive—and invasive—surveillance, including in the context of COVID-19 (Véliz, 2020). A number of authors stress the centrality of surveillance to public health efforts, as well as the potential risks of failure to surveil (e.g. Fairchild *et al.*, 2008: 30; Petrini, 2013; WHO, 2017: 10, 17; Gilbert *et al.*, 2019: 176; Lee, 2019: 320; Wood *et al.*, 2019) with Mello and Wang (2020: 951) suggesting that ‘the question is not whether to use new data sources—such as cellphones, wearables, video surveillance, social media, internet searches and news and crowd-sourced symptom self-reports—but how’. On the other hand, there are clear ethical issues involved in infectious disease surveillance, especially when it is opposed by many of those who are sufferers of the particular condition in question, as has been the case with HIV/AIDS in some jurisdictions (Fairchild, 2003; Fairchild *et al.*, 2008: 32–34; Klingler *et al.*, 2017: 1–2; Lee, 2019).

Some of the issues raised by infectious disease surveillance are ones which also affect the use of mHealth in the context of personal responsibility. A particularly obvious example is privacy, e.g. Lee (2019: 323). However, while there may be cases where a person is suitably responsible for having an infectious disease which is the subject of traditional infectious disease surveillance, the justification for surveillance in this case—preventing the spread of disease—is very different than the justification in cases of responsibilisation. Indeed, the primary justification for infectious disease surveillance offered in the literature is a broadly consequentialist one, presupposing a specifically health-related benefit which could not be achieved in other ways, and which outweighs potential harms (Fairchild *et al.*, 2008; Lee *et al.*, 2012: 38–42; Petrini and Ricciardi, 2015: 273; WHO, 2017). Even this is not universally accepted—for instance, Rubel (2012: 2) rejects justifications that reveal to an aggregate good, arguing that surveillance can be justified only if it protects ‘basic interests’—but in any case does not obviously apply to surveillance in the service of responsibilisation. Rather, the most obvious justification for responsibility-based surveillance would be *desert*-based, i.e. that those who are suitably responsible for their ill health ought to bear the burdens of it (financial or otherwise). Of course, one might also hope that a focus on personal responsibility will improve public health by disincentivising certain behaviours. Yet the central justification for infectious disease surveillance seems inimical to the idea of responsibilisation, with the WHO (2017: 46) reinforcing the idea that relevant data should not be used, nor given to those who would use it, to ‘take action against’ individuals.

Moreover, whereas infectious disease surveillance is typically focused on *aggregate* effects and on guiding public policy, personal health surveillance by necessity will involve a focus on individuals. Thus, while there are some clear parallels between existing infectious disease surveillance and ‘personal health surveillance’, the latter is a clearly distinct (potential) phenomenon that could not easily draw on the existence of the former for justification. Nonetheless, both types of surveillance may fall under the broad sphere of ‘public health’. Whereas infectious disease surveillance is more obviously concerned with public health, namely the targeting of public health policy, personal health surveillance may be concerned with a number of issues that are related to public health, including prevention of disease by disincentivising irresponsible behaviour, and the appropriate allocation of public health resources.

I return now to what I termed the ‘optimistic’ case for the use of mHealth technologies in healthcare responsibilisation:

*The state or an appropriate medical authority monitors whether patients are behaving in appropriate ways (e.g. taking moderate exercise) given their health needs or achieving certain health targets (e.g. reductions in cholesterol) without patients needing frequent, direct medical contact. Since patients have direct, quantifiable access to their health outcomes on a daily basis, they take greater responsibility for their health. Behavioural targets are more precise: for instance, rather than recommending that a patient take ‘regular, moderate exercise’, doctors can recommend more personalised targets, knowing that the patient can keep track. Previously opaque health outcomes are now available. A diabetic patient who might have sincerely believed they were keeping their blood sugars in control could only check whether this was accurate by regularly attending a medical appointment, which cannot occur every day (nor is it desirable that it should do so). The ability to self-monitor on a daily basis means that the patient now has more regular access to relevant information. This is both intrinsically desirable and removes one kind of excuse against responsibility for health outcomes, since patients cannot appeal to reasonable ignorance.*

For those who wish to use responsibility as a criterion for the allocation of healthcare (e.g. using responsibility as a tie-break when patients unavoidably compete for resources), the idea of mHealth may seem attractive. Our health is affected by choices we make in every aspect of our lives yet is subject to arguably more significant influences from our social and physical environment. Some unhealthy behaviours are thus either easier to detect, or more susceptible to being noticed for other reasons (e.g. because they are socially unpopular), than others. It is unfair if some people are penalised for choices that impact their health, while others make choices with similar impacts but face no penalty. Additionally, merely detecting a behaviour does not indicate its causes, e.g. whether patients engage in ‘unhealthy’ behaviour due to limited options.

In the absence of other evidence, the judgement about whether a patient is responsible for their ill health must depend on the patient’s own reports. Even without penalties, patients are sometimes reluctant to be open with doctors (Levy *et al.*, 2018). Penalties will presumably increase this tendency. Aside from undermining the evidence base for holding patients responsible, this will likely have a wider negative effect on the efficacy of treatment.

Surveillance might seem to mitigate both problems. Of course, only the most intrusive surveillance state could hope to fully eradicate the problem of detectability (and even this is doubtful). Responsibility for poor health, and various factors that might justify unhealthy behaviour, typically comes before any interaction with healthcare services. A comprehensively non-discriminatory system seemingly needs to surveil *all* individuals. Unhealthy behaviours do not occur only in public, nor can they always be detected after the fact. So, individuals would need to be surveilled at *all times* for the most comprehensive—and, thus, one might think, fairest—information about responsibility. For instance, Martani and Starke (2019: 252) consider the possibility of health providers forcing a choice to prospective patients between providing evidence that they are *not* relevantly responsible for their health needs, and rationing access.

This picture is deeply unattractive. Even if the citizenry of a country supports an increase in responsibilisation in healthcare for this reason, they may be unwilling to accept such comprehensive surveillance. Such a system would involve excessive capacity of government to *dominate* individuals (e.g. Pettit, 1997); an unwelcome increase in the political power of the state and its agents (e.g. Stahl, 2016); and would be excessively intrusive on citizens’ private lives (e.g. Lupton, 2012: 232, 239). Holding people responsible for their health is not of such urgency or necessity that the lack of a democratic mandate can be overruled. Even in the more limited context of employer surveillance of their employees via mHealth apps, significant concerns have been raised already, with Nissenbaum and Patterson (2016: 87) citing Stone’s (2002) objection to the establishment of ‘boundaryless workspaces’, and Selmi’s (2006: 1046) concern that ‘it is one thing to give an employer broad dominion over its own workplace but quite another to extend that dominion wherever the employee goes’.

While a democratic mandate is necessary for sanctioning such a programme of mass surveillance, it is not sufficient. While people disagree about the moral and political criteria for a justified surveillance programme, there is general agreement that widespread surveillance of the sort that covers an entire population must meet a standard of proportionality (Macnish, 2014; Rønn and Lippert-Rasmussen, 2020). Since even a well-intentioned surveillance programme, supported by a democratic majority, has the potential for significant abuse, the good that is acquired has to be significant. While some good might come out of comprehensive personal health surveillance, it seems unlikely to be sufficient to justify such sweeping oversight, even on an undemanding understanding of what proportionality requires (e.g. that the benefits incurred must only equal the costs, as opposed to significantly outweighing them).

## Purely ‘Health’ Surveillance?

Supporters of responsibilisation might object that the above discussion is fanciful: nobody wants complete acquiescence to a surveillance state. The problem, they might argue, is that such a state goes beyond *health* surveillance to the surveillance of every aspect of our lives. This invites the question of what surveillance that focused solely on health would look like. Carving out a distinctive sphere of ‘health’ is difficult (Segall, 2007; Wilson, 2009) and goods which do not seem to be primarily health-related may have greater effects on health than those behaviours and services which are commonly seen as belonging in the ‘health’ sphere (Marmot, 2005). One possible meaning of personal health surveillance is stipulative: surveillance is health-related when it monitors a health condition, or a behaviour that has been established *in that patient* to contribute to a health condition. For example, as someone with no diagnosed health conditions I can eat what I want, and it would be an unacceptable intrusion to monitor my health. If I were diagnosed with diabetes, it would be a legitimately *health*-related form of surveillance to monitor my diet and blood sugar levels. On this view, personal health surveillance is a reactive rather than preventive measure.

This response must accept a partial retreat on one of the two problems that personal health surveillance was supposed to solve. We can abandon the ambition to hold people responsible for health-affecting choices they make prior to entering the healthcare system. Alternatively, we must accept that due to a lack of surveillance, our evidence base for whether people are responsible for their ill health will often be based on self-reporting and easily observable behaviours. In either case, the issue of fairness re-emerges.

It is also not clear that even this reduced scope for state surveillance is proportionate, given the expected benefits. A personal health surveillance system backing up a policy of responsibility-based penalties would require that personal data were readily available to a much wider set of individuals than is currently normal. For instance, it would need to be transferred if the patient changed primary care doctor; it might need to be available in all national hospitals. Such a system, even restricted solely to personal health surveillance, routinely mistrusts patients, treating them as though they are either intentionally misleading the healthcare system or incapable of handling their own health adequately. It is therefore a system that risks demeaning patients, and turning ill health, which can already be a source of shame for various reasons, into a status of subjugation.

Moreover, different health issues will require different kinds of surveillance. For instance, if the behaviour for which the patient is to be held responsible is taking their daily medication, we might set up a pillbox that both prompts and records opening but does not surveil further activity. Such cases sit at one end of a spectrum of intrusiveness and may seem to be a reasonable level of surveillance. However, other behaviours seem to require almost constant surveillance. Consider a patient who is held responsible for engaging in a particular level of activity each day. We might begin with a pedometer, again a relatively unobtrusive form of surveillance. However, while taking a greater number of steps is probably better than a more sedentary lifestyle, merely taking a particular number of steps may not have a significant effect on health; for instance, if those steps fail to get one’s heart rate up. An effective surveillance system might therefore need to target patients’ vital signs. Finally, a widespread adoption of activity surveillance may well lead to some—perhaps many—individuals ‘gaming’ the system. Those who currently have a step counter on their phone, for instance, may know that the counter goes up not only if you walk or run somewhere, but also if you simply shake the phone. Insurers and governments might therefore decide that actual movement needs to be tracked as well as number of steps, taking advantage of the GPS capabilities that many phones have. In a climate of distrust, we have therefore quickly moved from a relatively low-level intrusion to a significant level of data collection.

## Surveillance as a Last Resort

In this section, I consider an even narrower scope for personal health surveillance, focusing on patients who repeatedly fail to meet minimal standards of responsibility for their health despite being capable of doing so. However, I also raise several problems with this proposal, both in this section and in the next.

The case for more limited personal health surveillance relies on the assumption that we are sometimes justified in giving additional burdens to those who are appropriately responsible for their care, e.g. by denying them care; setting their treatment as a lower priority relative to others; or imposing (additional) financial costs beyond what is standardly imposed. Recall that this article remains neutral on whether any of these are independently justified. Rather, the narrower version of personal health surveillance considered in this section involves using surveillance not as standard practice for all patients but a ‘Last Resort’ for patients who will otherwise legitimately incur one of the above-mentioned penalties due to their responsibility for their health needs.

The basic case for imposing penalties in such circumstances is that when patients could reasonably be expected to make choices that would improve their health (i.e. when it would not involve significant burdens in other areas of their lives, and when such choices are clearly explained and made available to them), but do not do so, they impose additional costs on the health care service, and hence on some of those who use and fund that service.

This case is highly controversial. Some deny that people can be responsible in a way that justifies such penalties (Sharkey and Gillam, 2010; Pereboom, 2014; Caruso, 2017). Others argue that whether or not this is conceptually possible, we are not able to detect such responsibility with sufficient accuracy (Shelton and Balint, 1997; Glantz, 2007; Friesen, 2018). I remind readers, however, that my approach in this article is to criticise the use of personal health surveillance to enforce responsibility *even if* proponents of responsibilisation can overcome these and other criticisms.

The policy of Last Resort might seem to have several advantages over the policies considered above. It does not place patients routinely under surveillance, and so is better placed with respect to proportionality. Since access to healthcare is a basic entitlement, there is no justification for placing conditions on access for patients who behave responsibly. However—despite being a basic entitlement—patients might plausibly be thought to have responsibilities as well as rights when it comes to accessing healthcare. Since the policy of Last Resort places conditions on access only for those who have already failed their responsibilities, an advocate might say, there is justification available for surveillance that is not available for more general policies. Precisely what the structure of this justification is depends on a more general argument about why it is legitimate to hold patients substantively responsible for their health. But in focusing on patients whose responsibility has already been reasonably established, Last Resort is better placed than similar policies with a wider scope to meet this justificatory burden. Moreover, the default approach is to trust patients, and to treat them as though they are entitled to the service they are using.

Yet this in itself raises a challenge. Recall that one putative attraction of personal health surveillance was to overcome epistemic barriers to determining patient responsibility. If we are justified in implementing surveillance only when patients have *already* reached a point where they have been deemed sufficiently responsible to face penalties, this problem remains. A policy of responsibilisation will need an alternative way of evidencing patient responsibility, reintroducing the problem of detection. Importantly, we cannot simply rely on patients’ doctors to relay whether they have been making reasonable efforts to remain healthy. While doctors clearly have some advantage in judging what is best for a patient, such a policy leaves far too much space for personal and systemic biases. For instance, various findings suggest that many medical professionals show bias in their treatment recommendations on the basis of sex and gender (Hamberg, 2008), ethnicity (Hoffman *et al.*, 2016) and whether a patient is perceived as ‘fat’ (Fruh *et al.*, 2016; Nath, 2019: 580). If medical professionals show bias in their treatment recommendations, there is clearly a risk that they will also show bias in making the (arguably vaguer) judgement about what steps it is ‘reasonable’ for a patient to take, including misidentifying the burdens particular activities will have on a patient. A reasonable process of nomination for Last Resort would therefore need to be more formalised and transparent than relying on doctors’ recommendations. It would also need to be open to a process of appeal that was not (financially or otherwise) inaccessible to patients. Aside from anything else, this challenges the thought that a surveillance programme would be a cost-saving exercise.

## Penalties and Fairness

Challenges of fairness arise whenever we select only some of a relevant class of individuals for benefit or penalty. Part of the answer to this challenge must be an admission that the problem of fairness arises in almost all attempts to hold large groups of people to standards of behaviour. In any widespread system, there will be false positives (people who are held responsible despite not being so) and false negatives (people whose responsible behaviour goes undetected). Nonetheless, when the system in question allocates something of such importance as healthcare, this answer is not enough: it must also be clear that incidences of these types of mistake are kept sufficiently low.

This challenge can be mitigated if we can show that although not all of the relevant class of individuals were correctly selected, the most significant cases were. For instance, the degree of justification for penalising people who are responsible for their own poor health seems to increase when they are more *reckless*, more *unreasonable*, or had *greater opportunity* to avoid the relevant behaviour (where this involves both the range of alternatives available to a person, and the ease with which those options can be chosen). A mechanism that picked out the most reckless, unreasonable and easily avoidable cases for penalty might thus be fair even if it did not pick out every case.

What would it take to focus on the most reckless or unreasonable cases? All else being equal, I assume that it is more unreasonable for someone to engage in a health-affecting behaviour if they have been offered support in avoiding that behaviour; if they have been warned of the health effects of the behaviour; and if avoiding the behaviour would have relatively few costs (Savulescu, 2018). While these are not the only ways of being unreasonably irresponsible, this does suggest that if a healthcare system provided such support and information, it might then be acceptable to hold patients substantively responsible.

Importantly, however, such a policy must take account of the personal circumstances of a patient. One of the most compelling objections to calls for responsibilisation is that they will tend to target those who are already vulnerable in society, and/or for whom adapting mandated behaviour changes will be particularly burdensome.^4^ It is essential to the fairness of holding patients responsible that the *difficulty* of adhering to particular habits and behaviours is recognised, and that it is acknowledged to vary depending on one’s circumstances. In addition, the *reasonableness* of failing to adopt certain healthy behaviours also varies depending on one’s circumstances, since health is not the only thing of value in our lives. Sometimes we rightly sacrifice health for other benefits, either for ourselves or others. Finally, recent work on the capacities required for moral responsibility has stressed the importance of seeing such capacities—e.g. the capacity to respond to moral reasons—as ‘relational … partly constituted by both agent and circumstance’ (Vargas, 2013: 206).^5^ A policy of responsibilisation must recognise the considerable role of social circumstances in determining people’s health, and the limits such circumstances place on a person’s ability to pursue ‘reasonable’ behaviours. It is possible to theoretically imagine a healthcare system that held people substantively responsible in this sensitive way, and we thus cannot rule out the idea of personal health surveillance on these grounds absolutely. Yet as a pragmatic objection, worries about insufficient differentiation of circumstance are significant. Particularly where personal health surveillance is pursued as a primarily cost-cutting exercise, we have reason to be sceptical about whether it is realistic to expect healthcare systems to properly account for such considerable differences in circumstances.

Personal health surveillance as a way of enforcing responsibility also introduces new issues. Consider two types of surveillance technologies, which correspond to the two functions of mHealth introduced in the above section, ‘Mobile Health, Surveillance and Two Challenges for Responsibilisation’. Behaviour-tracking technology would track users’ activities, assuming that particular behaviours increase the likelihood of desired health outcomes. To make such targets enforceable with penalties, we would need excellent evidence that they are both achievable and effective not only on average, but for the particular patient in question. The use of ‘generic’ targets that fail to take account of a patient’s personal circumstances and health needs raise issues of fairness where this leads to a patient being forced to adopt behavioural targets that are not appropriate for them, or not achievable in their personal circumstances. Consider, for example, the claim that many mobile phone-based pedometers do not accurately count steps when the user is pushing a pram, a complaint that many users have posted about online. The internet is full of ‘hacks’ to get around this problem, such as strapping the monitor to one’s ankle, and so it is not insurmountable. But there are more general issues raised by this example:

1. The manufacturers of the products did not consider a form of exercise that is common for many people, namely taking their child out for a walk.
2. The solution was not immediately obvious for many users, because many simply did not realise what the problem was.
3. The activity in question is one that, while certainly undertaken by men, is still more likely to be undertaken by women (given, for instance, the common disparities in social expectations about care, and legal allowance of parental leave). There is therefore an unintentional gender bias in the way these products track fitness. An uncritical adoption of similar technology in personal health surveillance would translate this bias to enforcement of responsibility.

On the other hand, outcome-tracking technologies offer more direct access to patients’ biological processes. Outcome-tracking might enforce responsibility by getting patients to self-monitor their health and take appropriate action when readings hit particular levels. Such technologies could be used for surveillance by reporting both the outcome-related data and whether the patient responds appropriately to readings which fall outside of their targets.

We should note the distinction between holding a patient responsible for responding appropriately to an off-target reading and making them responsible for bringing their biological readings back on target. While the former is still ultimately a form of behaviour that patients can adopt, the latter will often be out of the patient’s control; they might do everything they ought, and yet still fail to achieve their target. Even with this distinction in place, there is a risk that holding patients responsible for their biomedical states places too much burden on them. Although such data can be translated for the patient (‘If the reading is below 80, you need to take your medication'), holding patients responsible this way may increase reluctance to seek medical help because they may feel expected to ‘fix’ problems themselves; this may be particularly acute where patients know they will be penalised for failing to behave ‘appropriately’.

Personal health surveillance faces further ethical issues. One such issue, which has been central to academic and popular discussion of the ethics of mHealth quite generally, is privacy. Privacy can be understood in various ways, though Avancha *et al.* (2012) suggest that ‘control … is fundamental to privacy’, an idea echoed by Kotz (2011: 1), who says that ‘health information privacy is an individual’s right to control the acquisition, uses or disclosures of his or her identifiable data’. Privacy is an under-regulated element of mHealth, which as Martinez-Martin and Kreitmair (2018) suggest, is ‘a major concern when it comes to protecting the interests of users’, with ‘behavioural information … shared, stored and potentially sold to third parties’. Avancha *et al.* suggest several issues which are central to the regulation of privacy, including individual control over data; openness and transparency of those accessing and controlling data; and accountability for misuses of data, while also outlining various privacy-protection frameworks. Other, similar accounts can be found in Mendelson and Wolf (2017); Jusob *et al.* (2017); and Iwaya *et al.* (2018), while privacy as a concern for mHealth or health surveillance more generally is raised by Hendrix and Buck (2009: 482–499); Nissenbaum and Patterson (2016); Kreitmair *et al.* (2017); the WHO (2017: 37); Cvrkel (2018: 517); Kreitmair (2019: 158); Wood *et al.* (2019: 471); Lee (2019: 324–6); and Véliz (2020).

These broader concerns apply only partially to the case of Last Resort. Patients who are subject to Last Resort surveillance must by necessity have less control over who can access their data, and thus there is an inherent limit to their privacy rights compared with the typical mHealth user. Thus, it is not true that a patient operating under a scheme of Last Resort could have the typical right to decide precisely who has access to their health data. Yet there is still an onus on those who manage the relevant data to ensure that it is stored securely: wider access is not universal access. Patients under personal health surveillance cannot be treated as if their privacy does not matter. In addition, patients still have the right to know who has access to their data, under what circumstances, and why. As Mendelson and Wolf (2017: 5) note, there is even in non-punitive cases of the use of mHealth an ‘asymmetry of power’ between those whose data are accessed and those who access it. This asymmetry seems bound to be exacerbated when (the terms of) a patient’s access to care is on the line.

One way to think about this is in terms of ownership. Cvrkel (2018: 517) raises the question of who owns the data that is generated by users of mHealth apps. Assuming that our default answer is that the user should have at least *partial* ownership rights, there is no reason to think that patients who are covered by the Last Resort approach should *completely forego* ownership of their own data; rather, they simply have it limited in one way. Thus, even if patients who face the option of Last Resort have a reduced claim of control over particular forms of data (i.e. the data directly relevant to the health condition for which Last Resort is imposed), this does not mean that the treatment of patient data is straightforward. Consider a case where the relevant mHealth data involves tracking a patient’s movement (e.g. to ensure that they have done enough exercise). The most straightforward way to do this would be through an app on the patient’s mobile phone. Since mobile phones typically have one or more geolocation technologies, such tracking also raises the possibility of finding out other facts about the patient. As Carter *et al.* (2015) note, this information may include ‘where you live, where your children go to school, whether you visit a therapist and if so how often, how often you visit drinking or gambling establishments, whether you arrive early or late to work, whether you have participated in a protest or are associated with outlawed or terrorist organizations and other habits or routines’. This particular form of mHealth generates the possibility of patients being pressured into providing information they are not happy to share, and which has no direct relevance to the justification for surveilling them in the first place. The justification for placing someone under personal health surveillance on the Last Resort model is not that they have behaved in a way that undermines any right to privacy or autonomy, but that their actions have specific implications in one area of their life alone (see Sax, 2017, cited in Martani and Starke, 2019: 242). That someone has been placed under personal health surveillance as a Last Resort cannot be used to justify further, unrelated incursions on their rights.

A further issue with the use of some forms of mHealth for surveillance is the question of whether patients are adequately equipped to respond appropriately to data. We can imagine, for instance, a patient who is tasked with increasing exercise in order to reduce their percentage of body fat. The patient duly completes the required amount of exercise, but for whatever reason sees very little change in body fat percentage. If a patient has simply been left to deal with this information on their own, they may easily become demoralised, reducing their short-term motivation to continue exercising (Castelnuovo *et al.*, 2014; Lucivero and Jongsma, 2018: 687). Patients under personal health surveillance may therefore need to be provided with access to regular check-ins with doctors, medical counsellors or peer support networks (in person or through other forms of e-health), in order to put data into context, and to remind them that their targets are behavioural rather than outcome-focused.

It should be clear, then, that personal health surveillance, even as a last resort, cannot simply involve handing patients a device and some instructions. As Lucivero and Jongsma (2018: 686) put it, ‘despite the hype around mHealth, there are still many uncertainties around the safety, reliability and accuracy of mHealth systems’ (see also Martani *et al.*, 2019: 5; Martani and Starke, 2019: 256). Patients’ ability to meet targets and to interpret results, and their understanding of precisely what they have responsibility for, need to be carefully considered. In addition, we must be realistically confident that measurements provided by personal health surveillance technologies are accurate (DiStefano and Schmidt, 2016: 215). And even if patients allow their data to be accessed by a wider range of individuals than normal, the process of data storage and sharing must be both secure and transparent.

A final, practical problem with using mHealth technologies for surveillance is the ‘digital divide’ (Wood *et al.*, 2019: 472; Mello and Wang, 2020: 951). While some forms of mHealth involve giving patients specialised devices, others make use of existing devices such as smart phones. Yet some patients (Paldan *et al.*, 2018; Raber *et al.*, 2019) do not have access to these technologies. If some personal health surveillance relies on existing device ownership, we would face a choice between providing patients with the relevant technologies or excluding them from the opportunity to opt for personal health surveillance instead of exclusion. The former option reduces further the degree to which personal health surveillance represents a cost-saving exercise, while the latter option is clearly unjust, since it excludes people from a program of public healthcare provision based solely on wealth.

## Conclusions

My aim in this article has been to critically examine a view which is conditional on the moral acceptability of sometimes holding patients responsible for their health-related behaviours. Without endorsing such a view, I suggested that an under-explored issue with this approach is the problem of enforceability and detection, i.e. how we know when a patient has been behaving in the relevant ways. I suggested that, in the context of increasing use of mHealth technologies by employers and insurance companies to engage in ‘personal health surveillance’ against employees and clients, there is real potential for political states to begin exploring this option too.

However, I argued that while the increasing use of mHealth technologies may appear to present a solution to several problems facing those who wish to use responsibility as a rationing tool in healthcare, any plausible attempt to realise this faces significant ethical and practical problems of its own. The problem of fairness, related to detection, could only be solved by an unacceptably broad scope for personal health surveillance. Offering personal health surveillance as a last resort to patients who have already been judged suitably responsible for their health needs is a more plausible proposal, but still faces a range of ethical challenges. Thus, while mHealth technologies may appear to promise to ‘close the loop’ of enforceability when it comes to the responsibilisation of healthcare, in practice it faces considerable challenges.

## Footnotes

1 As far as I am aware, this is the only other work to consider this possibility directly; Martani and Starke offer distinct criticisms of this proposal, which are complementary to mine and with which I concur.

2 See Stemplowska (2009) for discussion.

3 My thanks to an anonymous referee for pointing out this gap in the original draft.

4 See Nath (2019) on the burdens some face in trying to lose weight.

5 See also McGeer (2015).
